# Performance and Wellbeing Research Priorities in Premiership Women's Rugby: A Delphi Study Including Players and Staff

**DOI:** 10.1002/ejsc.70102

**Published:** 2025-12-23

**Authors:** Omar Heyward, Sarah Whitehead, Gregory Roe, Holly Peace, Amy Walmsley, Simon Kemp, Ben Jones, Matt Cross, Terri Denham, Chaminda Goonetilleke, Gaby Halloran, Gareth Harris, David Holmes, Ken Kabongo, Steph McNally, Liam McStay, Sam MacGregor, Tom Oglethorpe, Keith Stokes

**Affiliations:** ^1^ Carnegie Applied Rugby Research (CARR) Centre Carnegie School of Sport Leeds Beckett University Leeds UK; ^2^ Rugby Football Union Twickenham UK; ^3^ Premiership Women's Rugby London UK; ^4^ London School of Hygiene and Tropical Medicine London UK; ^5^ England Performance Unit Rugby Football League Manchester UK; ^6^ Division of Physiological Sciences and Health Through Physical Activity, Lifestyle and Sport Research Centre, Department of Human Biology, Faculty of Health Sciences University of Cape Town Cape Town South Africa; ^7^ PREM Rugby London UK; ^8^ School of Behavioural and Health Sciences, Faculty of Health Sciences Australian Catholic University Brisbane Australia; ^9^ Leicester Tigers Leicester UK; ^10^ Trailfinders Women Ealing UK; ^11^ Saracens London UK; ^12^ Bristol Bears Bristol UK; ^13^ Sale Sharks Sale UK; ^14^ Exeter Chiefs Exeter UK; ^15^ Gloucester‐Hartpury Hartpury UK; ^16^ Loughborough Lightning Loughborough UK; ^17^ Harlequins London UK; ^18^ Centre for Health and Injury and Illness Prevention in Sport University of Bath Bath UK; ^19^ UK Collaborating Centre on Injury and Illness Prevention in Sport (UKCCIIS) University of Bath Bath UK

## Abstract

Women's sport has seen substantial growth in recent years, with increased attention to athlete performance and welfare. To support the ongoing professionalisation of women's rugby, performance and wellbeing must be prioritised. This study used a three‐round Delphi‐process to establish performance and wellbeing research priorities for Premiership Women's Rugby (PWR) in England. In Round 1, players and staff provided research priorities, which were grouped into higher‐order categories and themes via content analysis. In Rounds 2 and 3, participants ranked higher‐order categories on a 1–5 Likert scale. Consensus was defined as ≥ 70% agreement. Seventy‐seven participants responded in Round 1 (47 and 43 in Rounds 2 and 3). Player and staff experience of playing or working in PWR was 5.0 (2.0–7.0) and 2.5 (2.0–4.0) years. Following Round 1321 research priorities were provided, 32 higher‐order research priorities and 14 categories were identified, within three themes: performance, wellbeing and injury. Following Round 3, nine research priorities reached consensus within performance (*n* = 1), wellbeing (*n* = 4) and injury (*n* = 4). The highest rated priority was ‘*Investigate the impact of being a dual‐career athlete on wellbeing, and any* *support mechanisms required*’ (79%). Future research should prioritise studies which are feasible and currently lack a comprehensive evidence‐base. This will enable researchers and governing bodies to address relevant knowledge gaps and inform ongoing performance and player safety initiatives. The research priorities identified in this study, by PWR players and staff, could be investigated to support the development of women's rugby domestically. These findings may also be applicable to other women's sports and leagues globally.

## Highlights

- This study identified nine research priorities for Premiership Women's Rugby across performance, wellbeing and injury themes.
- The highest rated priority was to ‘*Investigate the impact of being a dual‐career athlete on wellbeing, and any support mechanisms required*’.
- This study captures the lived experiences and immediate concerns of women's rugby players, with 63% of participants being players. Wellbeing and injury‐related topics were prioritised over performance, reflecting evolving needs within a developing professional league.
- Findings provide a collaboratively derived research agenda to inform evidence‐based welfare and performance strategies in women's rugby and other women's sports.

## 1 Introduction

Women's rugby union has seen substantial growth in recent years, with an increase in both participation and professionalisation. In 2023, the global female playing population increased by seven percent to almost two million, with a 33.9% increase in active registered female players (World Rugby 2024). Meanwhile, at the elite level, there has been substantial investment with the launch of international competitions, such as the WXV, and semiprofessional competitions, for example, Premiership Women's Rugby (PWR). The PWR competition (formerly Premier 15 s) was established in 2023 to deliver a 10‐year strategy for women's rugby in England, with the aim to deliver the world's best women's rugby competition (Premiership Women’s Rugby 2024). To support the continued growth and professionalisation of women's rugby, there should be an ongoing focus on player welfare and performance alongside the focus on commercialisation (Saynor et al. 2024).

It is widely acknowledged that the evidence base from men's and male populations should not be applied to women's and female athletes due to gendered environments and sex‐related differences (Parsons et al. 2021). There has been a drive in recent years to close the sex and gender data gap in research and sport science and medicine support (Cowley et al. 2021; Emmonds et al. 2019; Saynor et al. 2024). There is emerging sport science and medicine evidence in women's rugby with 123 studies identified across the rugby codes (union, league and sevens) in 2021, 83% of which were published in the preceding decade (Heyward et al. 2022). The focus of research has predominately been on injury and physical performance (Heyward et al. 2022); however, there is still a call for further attention to female athlete health specific considerations in rugby (Moore et al. 2024). Although a biopsychosocial approach in sports medicine acknowledges the interaction between physical, psychological and social factors (McCormack et al. 2022; Truong et al. 2021), research in women's rugby is only beginning to adopt an intersectional perspective that recognises how multiple identities and contexts shape athlete experiences (Mkumbuzi 2024). Therefore, although it is evident that the sex‐specific (biological) and gender‐specific (sociocultural) research in women's rugby is on the rise, the appropriateness and effectiveness of the research need to be considered (Saynor et al. 2024).

To help guide future research in women's rugby, a systematic‐scoping review of all available evidence was conducted and presented to a global group of 31 elite players, sport scientists, clinicians and sports administrators. A Delphi consensus process was then undertaken to establish future research priorities (Heyward et al. 2021, 2022). Three research themes were identified as priorities: injury, physical performance and female health, with subthemes, such as the relationship between the menstrual cycle and injury, and long‐term health of women's rugby players, identified as being very high priorities (Heyward et al. 2022). Deriving research priorities in collaboration with sporting‐partners (e.g., players, coaches, practitioners and clinicians) is essential for the development of appropriate research questions and investment of resources (Hendricks 2021). In rapidly developing leagues, such as the PWR, consideration of the resources required to conduct high‐quality studies is particularly important, ensuring that research priorities are both relevant and feasible to implement. By involving sporting‐partners in the development of agendas, one of the perceived barriers to evidence‐based practice, the lack of relevant and applicable research (Fullagar et al. 2019; Jones et al. 2017), could be mitigated. Similarly, incorporating input from athletes can contribute to an athlete‐centred approach, recognising that different players may have different experiences and priorities, which can help guide research to better meet their needs (McCleery et al. 2024).

Recent research has utilised sporting‐partner panels via a Delphi approach to reach consensus on research priorities and agendas for specific organisations (e.g., Team USA (McCleery et al. 2024)), men's Premiership Rugby (Jones et al. 2025). As part of the 10‐year PWR strategy, there is commitment from the league and the governing body (Rugby Football Union [RFU]) to optimise player welfare and performance. The PWR manages the day‐to‐day operations of the league under RFU regulations. The two organisations collaborate on strategic performance and welfare initiatives. The research priorities previously generated for women's rugby globally (Heyward et al. 2022) may not have captured the specific needs and contextual factors of the PWR. The PWR is emerging as the most professional women's rugby union competition regarding structure, governance and resource and is therefore potentially unique compared to other women's rugby settings. To support the determination of an appropriate research agenda specific for the PWR, the perspectives of players and staff involved in this competition should be established. Therefore, this study aimed to replicate the methods used in the research priorities for men's Premiership Rugby study (Jones et al. 2025) and generate performance and wellbeing research priorities, specifically for women's rugby players from the perspectives of players and staff within PWR via a Delphi consensus method.

## 2 Method

### 2.1 Research Philosophy

This study was underpinned by pragmatism and therefore undertaken with the intention of producing practically meaningful knowledge. Pragmatic research should be focused on generating knowledge that provides practical solutions to an a priori identified problem (Morgan 2013). Unlike strict research paradigms (e.g., positivism or constructivism), pragmatism does not mandate a single fixed ontology or epistemology; rather, it views ‘truth’ in terms of what works to address the question at hand (Morgan 2013). Consequently, researchers place their questions and the practical problem at the centre of the design, and then select whatever methods—qualitative, quantitative or mixed—are most suited to generating useful solutions.

As research priorities have been identified for men's Premiership Rugby (Jones et al. 2025), the present study aimed to generate insights to develop research priorities based on the perspectives of PWR players and staff. To do so, a Delphi method was employed, following the ACCORD guidelines and recommendations of Jünger and colleagues (Gattrell et al. 2024; Jünger et al. 2017). Additionally, content analysis was implemented as a process of identifying patterns within the perspectives provided by the participants (Elo and Kyngäs 2008).

### 2.2 Study Design

A three‐round Delphi process (Diamond et al. 2014; Jünger et al. 2017; McMillan et al. 2016) was used to establish performance and wellbeing research priorities for PWR players. This study was not prospectively registered. Round 1 of the Delphi involved participants completing an online survey, in which they were asked to provide up to 10 research priorities for PWR in the areas of player performance (i.e., anything that contributes to how well a player or team can compete in match‐play) and wellbeing (i.e., a positive state and essential daily resource influenced by social, economic, and environmental factors, encompassing quality of life, health, and an individual's emotional, mental, social, and physical functioning) (Giles et al. 2020; Nutbeam and Muscat 2021). These were subsequently grouped into higher‐order categories and themes via content analysis (Elo and Kyngäs 2008). In Rounds 2 and 3 of the Delphi process, additional online surveys were provided in which participants were asked to rank the higher‐order categories from very low (i.e., 1) to very high priority (i.e., 5) on a 1–5 Likert scale. Consensus was defined as ≥ 70% agreement (Heyward et al. 2022; Jones et al. 2025). The study received institutional ethics approval (no. 136411), and all participants provided informed consent and participated voluntarily.

### 2.3 Participants

Players and staff at the nine PWR clubs were invited to participate alongside staff of the England Senior Women's team. To ensure that participants had adequate knowledge of PWR, staff were required to have a minimum of 1 year of experience working at PWR level or currently working at the international level (i.e., England Senior Women). Additionally, players were required to have a minimum of 3 years of experience playing at PWR level or have been selected in a match‐day squad at the international level (any country) at least once, combined with a minimum of 1 year of experience playing at PWR level. These criteria differ from those used in the men's Premiership Rugby study (Jones et al. 2025) to reflect the earlier stage of development of the PWR competition (e.g., men's Premiership Rugby staff and players were required to have a higher minimum number of years' experience [3 and 5 years]). Criteria were reviewed and agreed by the Women's Medical Advisory Group of the RFU and PWR to ensure the perspectives of sporting‐partners were reflected. Participants were classified into performance (e.g., strength and conditioning and nutritionist), medical (e.g., physiotherapist and medical doctor), coaching (e.g., rugby coach), administrative (e.g., team manager) or player categories.

### 2.4 Delphi Technique

#### 2.4.1 Round 1: Establishing Research Priorities in Premiership Women's Rugby

Prior to initiation, all teams were contacted by the RFU and provided with project details and a request to nominate a staff project lead (the individual responsible for coordinating the research within their respective team). Once the project leads were appointed, they were briefed on the purpose of the project and given an opportunity to ask questions via an online meeting. Following the meeting they were sent an email containing the survey, for distribution to players and staff within their team who met the inclusion criteria.

Each participant that met the inclusion criteria received an online survey (Supporting Information S1) hosted by Qualtrics Inc. (USA). In Section 1, the survey included questions including contact and demographic information (job role/playing position, ethnicity, years' experience). In Section 2, participants were asked to provide up to 10 research priorities in the areas of player wellbeing and performance. Definitions for wellbeing and performance were provided (Giles et al. 2020; Nutbeam and Muscat 2021), along with an example for each research priority, as per (Jones et al. 2025). Participants were given 9 weeks to respond to Round 1, with reminders from club research representatives to encourage participation.

### 2.5 Data Analysis

Inductive content analysis was used to group the research priorities provided by the participants in Round 1 into higher‐order categories and themes (Elo and Kyngäs 2008). Two authors (OH and SW) thoroughly familiarised themselves in the responses. Data were independently coded by OH using the process of abstraction (i.e., generation of categories). Sub‐categories, categories and finally, themes, that shared common features, were generated (Elo and Kyngäs 2008). Authors OH and SW discussed the results until both were satisfied that the subcategories (higher‐order research priorities), categories (groupings of higher‐order research priorities) and themes (groupings of categories) accurately represented the raw data. All members of the steering group (OH, HP, AW, BJ and KS) agreed on the subcategories, categories and themes.

#### 2.5.1 Rounds 2 and 3: Establishing Consensus on Research Priorities in Premiership Women's Rugby

##### 2.5.1.1 Round 2

A second online survey (Qualtrics Inc., USA) containing a table of the previously established research priorities for women's rugby globally and a link to the study (Heyward et al. 2022) were presented. These data were presented in Round 2 to reduce the risk of biasing participant responses in Round 1. Participants were also asked to review the subcategories, categories and themes generated from Round 1 in the present study and given an opportunity to suggest any additional research priorities.

Participants were subsequently asked to rank each of the higher‐order research priorities established in Round 1 on a Likert scale of 1–5 (1: very low priority, 2: low priority, 3: medium priority, 4: high priority and 5: very high priority) (Heyward et al. 2022; Jones et al. 2025). Participants were given 6 weeks to respond to the survey and were sent reminders to encourage participation.

To assess consensus, Likert scale responses were combined to form 3 agreement categories (i.e., low priority: 1 and 2, medium priority: 3 and high priority: 4 and 5) (Heyward et al. 2022; Jones et al. 2025). Descriptive statistics for the median and interquartile range were calculated. Consensus was defined as ≥ 70% agreement (Heyward et al. 2022; Jones et al. 2025; Zambaldi et al. 2017).

##### 2.5.1.2 Round 3

In Round 3, all participants (i.e., those who responded to Round 1) were provided feedback regarding the results via a third online survey (Qualtrics Inc., USA). This included a list of the higher‐order research priorities that reached consensus and a further list of those that did not reach consensus with the accompanying median for each respective response. Participants were invited to reflect on their previous rating and, if they wished, re‐rate any higher‐order research priorities that did not reach consensus in Round 2. Participants were given 4 weeks to respond and were sent reminders to encourage participation.

## 3 Results

### 3.1 Participants

Seventy‐seven participants responded in Round 1, of which 47 and 43 responded in Rounds 2 and 3, respectively. Participants represented players and (performance, medical, coaching and administration) staff (Table 1). Staff characteristics were as follows: sex; 42% female and 58% male, seasons experience working in PWR and/or England Senior Women; 2.5 [2.0–4.0] (median [interquartile range]), seasons experience working in another sport; 4.5 [2.0–7.8], education; 58% master’s degree, 21% undergraduate degree and 21% other (postgraduate certification and Advanced highers) and ethnic origin; 84% White, 11% Asian and 5% Mixed. Player characteristics were as follows: sex; 100% female, seasons playing in PWR; 5.0 [2.0–7.0], seasons playing in another senior league; 2.0 [0.0–4.0], number of times selected in pathway or senior international rugby union match‐day squad; 13.5 [5.3–30.8], education; 2% PhD, 26% Master’s degree, 55% undergraduate degree and 17% other (advanced‐levels and General Certificate of Secondary Education) and ethnic origin; 83% White, 7% Mixed, 5% Asian, 3% Black/African/Caribbean and 2% Other.

**TABLE 1:** Role and sex of participants by Delphi round.

	Round 1		Round 2		Round 3	
	*n*	%	*n*	%	*n*	%
Staff						
Performance	8	10	6	13	6	14
Medical	7	9	6	13	6	14
Coaching	2	3	2	4	2	5
Administration	2	3	2	4	2	5
Total	19	25	16	34	16	37
Player position						
Forward	33	43	17	36	14	33
Back	25	32	14	30	13	30
Total	58	75	31	66	27	63
Sex						
Female	66	86	37	79	34	79
Male	11	14	10	21	9	21
Total	77	100	47	100	43	100
Total participants	77	100	47	100	43	100

### 3.2 Round 1: Establishing Research Priorities in Premiership Women's Rugby

Participants initially provided a total of 321 research priorities (performance *n* = 168 and wellbeing *n* = 153). Removing duplicates and streamlining responses for clarity resulted in 32 unique higher‐order research priorities grouped into 14 categories. Furthermore, although participants were asked to provide performance or wellbeing research priorities, an additional theme of ‘injury’ was identified. This approach was undertaken due to the frequency and specificity of injury related responses as well as their relevance across both wellbeing and performance (Jones et al. 2025).

### 3.3 Rounds 2 and 3: Establishing Consensus on Research Priorities in Premiership Women's Rugby

In Round 2, an additional four higher‐order research priorities were identified to be included by participants, which resulted in a total of 36 priorities (wellbeing *n* = 13, performance *n* = 13 and injury *n* = 10). The additional four higher‐order research priorities were not identified in the previously established research priorities for women's rugby globally (Heyward et al. 2022). In Round 2, six higher‐order research priorities reached consensus (denoted by ‘a’ in Table 2). In Round 3, an additional three higher‐order research priorities reached consensus (Table 2). Higher‐order research themes that did not reach consensus are presented in Supporting Information S1: Table S1.

**TABLE 2:** Higher‐order research priorities that reached consensus, distribution of votes for priority across three categories and the median priority (interquartile range, IQR).

Category	Higher‐order research priority	Low priority (%)	Medium priority (%)	High priority (%)	Median priority (IQR)
Wellbeing theme					
External stressors	^a^Investigate the impact of being a dual‐career athlete on wellbeing and any support mechanisms required.	11	11	79	4.0 (4.0–5.0)
Medical	Investigation of the causes, prevalence, severity and management of mental health symptoms (e.g., emotional exhaustion) and disorders (e.g., depression and body dysmorphic disorder) and any associated support mechanisms required.	12	16	72	4.0 (3.0–4.5)
Psychology	Investigate the impact of high training loads or condensed season on wellbeing.	9	21	70	4.0 (3.0–5.0)
External stressors	Explore the impact of financial status on player wellbeing.	14	16	70	4.0 (3.0–4.0)
Performance theme					
Female‐specific	^a^Investigate the association between menstrual cycle status (e.g., phase, irregularity, hormonal contraceptive use and symptoms) and performance.	9	15	77	4.0 (4.0–5.0)
Injury theme					
Injury risk	^a^Explore return‐to‐play processes (e.g., post injury, concussion and childbirth) and subsequent injury risk and performance.	6	17	77	4.0 (4.0–5.0)
Injury risk	^a^Investigate the association between menstrual cycle status (e.g., phase, irregularity, hormonal contraceptive use and symptoms) and injury.	2	21	77	4.0 (4.0–5.0)
Injury risk reduction	^a^Investigate the effectiveness of targeted interventions (e.g. neck, shoulder capacity and ACL screening/intervention) for reducing injuries.	7	21	72	4.0 (3.0–4.0)
Injury risk	^a^Investigate the association between pitch surface and changing surfaces on injury.	13	17	70	4.0 (3.0–5.0)

^a^ Denotes consensus was achieved in Round 2. Priority rating: low (1‐very low and 2‐low), medium (3‐medium) and high (4‐high, 5‐very high). Percentages may not total 100 due to rounding.

## 4 Discussion

This study aimed to generate performance and wellbeing research priorities from the perspectives of PWR players and staff via methods used to identify research priorities in men's Premiership Rugby. Following the Delphi method to achieve consensus, nine research priorities were identified (wellbeing *n* = 4, performance *n* = 1 and injury *n* = 4). All priorities that reached consensus received a median priority of ‘high’ (i.e., 4 out of 5). The highest rated priority was 79% for ‘*Investigate the impact of being a dual‐career athlete on wellbeing, and any* *support mechanisms required*’, in the theme of Wellbeing. In comparison, 21 research priorities (wellbeing *n* = 6, performance *n* = 7 and injury *n* = 8) were identified for men's Premiership Rugby, with *‘Targeted interventions for reducing common injuries in the premiership’* as the highest rated (89%) priority (Jones et al. 2025). Although both studies used similar methods and a comparable number of Round 1 initial priorities (men's = 391 and women's = 321), differences in participant characteristics may explain the fewer priorities reaching consensus in the present study. For example, the men's study included a smaller proportion of players (6% vs. 63%), which may have influenced how topics were rated and prioritised. The present study highlights potential areas of future research focus as perceived by PWR players and staff. The findings of this study have relevance to PWR and may be relevant to other women's sports and leagues globally.

The PWR is the most established women's rugby union competition globally and is on a 10‐year strategic journey towards full professionalisation (Premiership Women’s Rugby 2024). Despite this progress, many PWR players are dual‐career athletes who rely on employment outside of rugby to financially support their sporting careers. Although diversity in employment status was not collected in this study, it is important to recognise that within the league there is considerable variation, including full‐time contracted players with no additional income, full‐time contracted players with additional income, part‐time contracted players who also study and/or work, players who are employed or studying full‐time outside of rugby, players who are employed or studying part‐time outside of rugby and unemployed players (Todd 2025). Dual‐careers are common among elite female athletes globally, including those competing in top‐tier competitions (e.g., Australian Football League Women's (Australia), Netball Super League (England) and Frauen‐Bundesliga (Germany)). As a result, dual‐career athlete investigations are relevant not only for PWR but also for many other women's sports leagues. Recent wellbeing‐related evidence on dual‐career athletes has investigated mental health (Aalto et al. 2024; Kegelaers et al. 2024). Kegelaers et al. (2024) identified that most studies indicated dual‐career athletes to have similar or decreased risk for mental health problems when compared to nondual career cohorts, which may reflect the identity beyond sport and psychological buffer that additional employment provides. In European sports, further evidence of athlete characterisation (e.g. (McKay et al. 2021)), competencies (e.g., resilience) and a focus on longitudinal studies of dual‐career athlete mental health are required (Aalto et al. 2024). These reviews highlight the potential benefits of dual‐career pathways and suggest that further investigations that account for sport‐specific and sociocultural contexts may be warranted.

The present study identified some key similarities and differences in research priorities when compared to global women's rugby research priorities (Heyward et al. 2022). Heyward et al. (2022) conducted a Delphi study to identify sports science and medicine research priorities in women's rugby. The study included a global group of 31 women's rugby experts that spanned multiple governing bodies and areas of expertise (i.e., elite player, sports science, clinician and administrator) who reviewed every available study in women's rugby. The priorities in the present study that aligned to the global research priorities were related to investigating mental health symptoms and disorders, injury risk reduction interventions and menstrual cycle status relationships with injury or performance. The prevalence of mental health symptoms and disorders in current male and female elite athletes ranges from 19% to 34% (Gouttebarge et al. 2019). However, further research on female athletes is needed as the meta‐analysis by Gouttebarge et al. (2019) included only one study that was conducted exclusively on female athletes. Match injury rates in elite women's rugby remains a concern, with a reported match injury incidence as high as 61 per 1000 h, and concussion reported as the most common injury (Starling et al. 2023; Williams 2024). As the tackle is the most injurious event in women's rugby (Starling et al. 2023), injury risk reduction strategies focused on tackle safety could play a role in reducing injury burden. The prioritisation of menstrual cycle relationships with injury or performance may reflect the increased media attention these topics have received (BBC Sport 2024). However, the current evidence on these topics is insufficient to inform any decision‐making in this area (Sale et al. 2023). To address the gap, large‐scale multisite projects collecting high‐quality data are required in to appropriately investigate the complex relationships between menstrual cycle, performance and injury in female athletes (Sale et al. 2023).

The present study identified research priorities within both the Wellbeing and Injury themes that were not identified in the global women's rugby consensus. The Wellbeing priorities in the present study were related to investigating dual‐career athletes, high training load or condensed season financial status. The injury priorities in the present study were related to return‐to‐play processes and pitch surface. The differences between priorities identified by PWR players and staff compared to those highlighted by global experts may reflect several factors, including contextual influences and demographic differences, within the respective cohorts. In terms of contextual factors, this study was conducted during a Women's Rugby World Cup year. Consequently, the PWR season was shorter than usual (6 months in 2024/25 compared to 9 months in 2023/24), which may have contributed towards prioritisation of condensed seasons and workload concerns. Regarding return‐to‐play processes, specific postpartum considerations have recently been published for women's rugby (Donnelly et al. 2024). As these recommendations are yet to be evidenced, they may have not yet been widely disseminated to PWR players and staff, potentially contributing to the prioritisation of this topic. Demographic differences between the present study and the global consensus process may also explain the divergence in research priorities. Participants in the present study were predominantly players (63%–75%), most of whom were well educated (2% held a PhD, 26% a master’s degree and 55% an undergraduate degree). In contrast, the global expert panel included sports scientists, clinicians, sports administrators and elite players with a minimum of national‐level experience. Additionally, players included in the global expert panel were required to have expertise in sport science or medicine (e.g., PhD and medical doctor). These demographic differences suggest that the present study captured the athlete voices, reflecting on the lived experiences and immediate concerns of players, whereas the global expert group was positioned to identify broader, evidence‐based gaps within the field.

The coconstruction of higher‐order research priorities in this study was supported by a diverse group of participants, including players, performance staff, medical personnel, coaches and administrative staff. A key strength of this work was the engagement of the end‐users, particularly the players, in the construction of research priorities. Notably, women's rugby players accounted for 63% of all participants in Round 3. This study contributes to addressing the sex and gender data gap in sport science and medicine (Cowley et al. 2021; Jones et al. 2025) and supports the development of approaches to enhance player safety and performance research in women's rugby (Saynor et al. 2024). Importantly, 79% of participants in Round 3 were women (Table 1), ensuring that their lived experiences were central to the development of research priorities.

This study is not without its limitations. Although the participant group included a range of player and staff roles, representation across ethnicities was limited, which is a known challenge that the RFU is actively addressing (England Rugby 2020). Additionally, although players identified topics they perceived as important, this does not necessarily confirm these as research priorities.

As research is not typically designed for players to engage with directly, some participants may not have had sufficient understanding of the existing evidence base to fully inform their responses during the Delphi process. To mitigate this, participants were provided with example research priorities in Round 1; however, this could have biased some responses. Additionally, a summary of the global women's rugby sport science and medicine research priorities (Heyward et al. 2022) and a study link were provided to participants during Round 2, although it remains unclear how accessible this was to players who may be less familiar with research.

As Round 3 was distributed to all participants from Round 1, it is possible that some individuals did not engage in every round of the Delphi process. Moreover, the relatively low representation of coaching and administrative staff may limit the generalisability of the findings to all coaches and administrators across the league.

Finally, only one performance‐related priority reached consensus, which contrasts to the global women's rugby consensus identifying physical performance as a high priority (Heyward et al. 2022). This difference may indicate that players in the present study placed greater importance on their wellbeing. It is also acknowledged that some of the injury‐related priorities have relevance to both wellbeing and performance. Despite these limitations, the PWR is recognised as the most established women's rugby union league globally, and therefore, some of the priorities identified in this study may have relevance and transferability to other women's sports leagues, both within rugby and in other sports. Common aspects, such as developing professionalisation, limited medical and performance resources and the prevalence of dual‐career athletes, may allow for meaningful transfer of findings across contexts (Emmonds et al. 2019).

## 5 Conclusion

This study aimed to establish research priorities for the PWR from the perspective of players and staff. A total of nine research priorities reached consensus across the wellbeing (*n* = 4), performance (*n* = 1) and injury (*n* = 4) themes. The highest‐rated priority was for ‘*Investigate the impact of being a dual‐career athlete on wellbeing, and any* *support mechanisms required*’ (79%). It is important that researchers remain cognisant of the resources required to conduct high‐quality studies on complex topics such as menstrual cycle, injury and performance relationships. As such, prioritising feasible research areas that currently lack a comprehensive evidence‐base can enable researchers and governing bodies to address relevant knowledge gaps and inform ongoing performance and player safety initiatives.

## Funding

The authors have nothing to report.

## Conflicts of Interest

All authors except SW and GR are employed by professional rugby clubs, professional leagues or governing bodies, therefore could be perceived to have a conflict of interest.

## Supporting information


Supporting Information S1

## References

[ejsc70102-bib-0001] Aalto, E. P. , J. Pons , S. Alcaraz , R. Zamora‐Solé , and Y. Ramis . 2024. “Psychological and Social Factors Associated With Mental Health of European Dual Career Athletes: A Systematic Review.” European Journal of Sport Science 24, no. 12: 1844–1864. 10.1002/ejsc.12218. 39533759 PMC11621382

[ejsc70102-bib-0002] BBC Sport . 2024. WSL Study Finds Injuries More Likely at Points in Menstrual Cycle. BBC Sport. Accessed, June 30, 2025. https://www.bbc.com/sport/articles/ck5k9l8z13wo.

[ejsc70102-bib-0003] Cowley, E. S. , A. A. Olenick , K. L. McNulty , and E. Z. Ross . 2021. “‘Invisible Sportswomen’: The Sex Data Gap in Sport and Exercise Science Research.” Women in Sport & Physical Activity Journal, September 29, no. 2: 146–151. 10.1123/wspaj.2021-0028.

[ejsc70102-bib-0004] Diamond, I. R. , R. C. Grant , B. M. Feldman , et al. 2014. “Defining Consensus: A Systematic Review Recommends Methodologic Criteria for Reporting of Delphi Studies.” Journal of Clinical Epidemiology, April 67, no. 4: 401–409. 10.1016/j.jclinepi.2013.12.002. 24581294

[ejsc70102-bib-0005] Donnelly, G. , C. Coltman , K. Dane , et al. 2024. “Prioritise Safety, Optimise Success! Return to Rugby Postpartum.” European Journal of Sport Science 24, no. 12: 1701–1718. 10.1002/ejsc.12144. 38978338 PMC11621390

[ejsc70102-bib-0006] Elo, S. , and H. Kyngäs . 2008. “The Qualitative Content Analysis Process.” Journal of Advanced Nursing, April 62, no. 1: 107–115. 10.1111/j.1365-2648.2007.04569.x. 18352969

[ejsc70102-bib-0007] Emmonds, S. , O. Heyward , and B. Jones . 2019. “The Challenge of Applying and Undertaking Research in Female Sport.” Sports Medicine ‐ Open 5, no. 1: 51. 10.1186/s40798-019-0224-x. 31832880 PMC6908527

[ejsc70102-bib-0008] England Rugby . 2020. “RFU Committed to Improving Diversity and Inclusion | Rugby Football Union.” Accessed October 30, 2025. https://www.englandrugby.com/follow/news‐and‐media/rfu‐committed‐to‐improving‐diversity‐and‐inclusion.

[ejsc70102-bib-0009] Fullagar, H. H. K. , A. McCall , F. M. Impellizzeri , T. Favero , and A. J. Coutts . 2019. “The Translation of Sport Science Research to the Field: A Current Opinion and Overview on the Perceptions of Practitioners, Researchers and Coaches.” Sports Medicine 49, no. 12: 1817–1824. 10.1007/s40279-019-01139-0. 31214978

[ejsc70102-bib-0010] Gattrell, W. T. , P. Logullo , E. J. van Zuuren , et al. 2024. “ACCORD (Accurate Consensus Reporting Document): A Reporting Guideline for Consensus Methods in Biomedicine Developed via a Modified Delphi.” PLoS Medicine, January 21, no. 1: e1004326. 10.1371/journal.pmed.1004326. 38261576 PMC10805282

[ejsc70102-bib-0011] Giles, S. , D. Fletcher , R. Arnold , A. Ashfield , and J. Harrison . 2020. “Measuring Well‐Being in Sport Performers: Where Are We now and How Do We Progress?” Sports Medicine, July 50, no. 7: 1255–1270. 10.1007/s40279-020-01274-z. 32103451 PMC7305091

[ejsc70102-bib-0012] Gouttebarge, V. , J. M. Castaldelli‐Maia , P. Gorczynski , et al. 2019. “Occurrence of Mental Health Symptoms and Disorders in Current and Former Elite Athletes: A Systematic Review and Meta‐Analysis.” British Journal of Sports Medicine, June 53, no. 11: 700–706. 10.1136/bjsports-2019-100671. 31097451 PMC6579497

[ejsc70102-bib-0013] Hendricks, S. 2021. “Rethinking Innovation and the Role of Stakeholder Engagement in Sport and Exercise Medicine.” BMJ Open Sport & Exercise Medicine, May 7, no. 2: e001009. 10.1136/bmjsem-2020-001009. PMC8160180 34123408

[ejsc70102-bib-0014] Heyward, O. , S. Emmonds , G. Roe , S. Scantlebury , K. Stokes and B. Jones 2021. “Applied Sport Science and Medicine of Women’s Rugby Codes: A Systematic‐Scoping Review and Consensus on Future Research Priorities Protocol.” BMJ Open Sport and Exercise Medicine, July 7, no. 3: e001108. 10.1136/bmjsem-2021-001108. PMC8317073 34394953

[ejsc70102-bib-0015] Heyward, O. , S. Emmonds , G. Roe , S. Scantlebury , K. Stokes , and B. Jones . 2022. “Applied Sports Science and Sports Medicine in Women’s Rugby: Systematic Scoping Review and Delphi Study to Establish Future Research Priorities.” BMJ Open Sport and Exercise Medicine 8, no. 3: e001287. 10.1136/bmjsem-2021-001287. PMC9310180 35979431

[ejsc70102-bib-0016] Jones, B. , O. Heyward , M. Cross , et al. 2025. “We’re all (Cauliflower) Ears’: A Delphi Study Including Staff and Players to Co‐Construct Sports Science and Medicine (Performance and Wellbeing) Research Priorities for Premiership Rugby.” European Journal of Sport Science 25, no. 7: e70007. 10.1002/ejsc.70007. 40600778 PMC12217044

[ejsc70102-bib-0017] Jones, B. , K. Till , S. Emmonds , et al. 2017. “Accessing Off‐Field Brains in Sport; An Applied Research Model to Develop Practice.” British Journal of Sports Medicine, July 53, no. 13: 791–793. 10.1136/bjsports-2016-097082. 28818959

[ejsc70102-bib-0018] Jünger, S. , S. A. Payne , J. Brine , L. Radbruch , and S. G. Brearley . 2017. “Guidance on Conducting and Reporting Delphi Studies (CREDES) in Palliative Care: Recommendations Based on a Methodological Systematic.” Review 31, no. 8: 684–706. 10.1177/0269216317690685. 28190381

[ejsc70102-bib-0019] Kegelaers, J. , P. Wylleman , S. Defruyt , et al. 2024. “The Mental Health of Student‐Athletes: A Systematic Scoping Review.” International Review of Sport and Exercise Psychology, December 17, no. 2: 848–881. 10.1080/1750984X.2022.2095657.

[ejsc70102-bib-0020] McCleery, J. , E. Diamond , R. Kelly , et al. 2024. “Centering the Female Athlete Voice in a Sports Science Research Agenda: A Modified Delphi Survey With Team USA Athletes.” British Journal of Sports Medicine, October 58, no. 19: 1107–1114. 10.1136/bjsports-2023-107886. 38981661 PMC11503037

[ejsc70102-bib-0021] McCormack, S. , K. Till , J. Wenlock , et al. 2022. “Contributors to Negative Biopsychosocial Health or Performance Outcomes in Rugby Players (Conbo): A Systematic Review and Delphi Study Protocol.” BMJ Open Sport & Exercise Medicine, October 8, no. 4: e001440. 10.1136/bmjsem-2022-001440. PMC9557262 36249486

[ejsc70102-bib-0022] McKay, A. K. A. , T. Stellingwerff , E. S. Smith , et al. 2021. “Defining Training and Performance Caliber: A Participant Classification Framework.” International Journal of Sports Physiology and Performance, December 17, no. 2: 317–331. 10.1123/ijspp.2021-0451. 34965513

[ejsc70102-bib-0023] McMillan, S. S. , M. King , and M. P. Tully . 2016. “How to Use the Nominal Group and Delphi Techniques.” International Journal of Clinical Pharmacy 38, no. 3: 655–662. 10.1007/s11096-016-0257-x. 26846316 PMC4909789

[ejsc70102-bib-0024] Mkumbuzi, N. S. 2024. “Women’s Rugby for All: Toward an Intersectional Women’s Rugby Research Agenda.” European Journal of Sport Science 24, no. 12: 1754–1764. 10.1002/ejsc.12127. 38874753 PMC11621387

[ejsc70102-bib-0025] Moore, I. S. , M. McCarthy‐Ryan , D. Palmer , J. Perkins , and E. Verhagen . 2024. “Is your System Fit for Purpose? Female Athlete Health Considerations for Rugby Injury and Illness Surveillance Systems.” European Journal of Sport Science 24, no. 12: 1688–1700. 10.1002/ejsc.12089. 39639642 PMC11621385

[ejsc70102-bib-0026] Morgan, D. L. 2013. Integrating Qualitative and Quantitative Methods: A Pragmatic Approach. Sage publications. Accessed 20 May 2025]. https://books.google.co.uk/books?hl=en&lr=&id=8r6wBAAAQBAJ&oi=fnd&pg=PP1&dq=Morgan+DL.+Integrating+Qualitative+and+Quantitative+Methods:+A+Pragmatic+Approach.+SAGE+Publications,+Inc.+2014.&ots=w2NVYIkSGe&sig=tTq5qrV3Xb2hre45RScqVY3EYE0.

[ejsc70102-bib-0027] Nutbeam, D. , and D. M. Muscat . 2021. “Health Promotion Glossary 2021.” Health Promotion International 36, no. 6: 1578–1598. 10.1093/heapro/daaa157. 33822939

[ejsc70102-bib-0028] Parsons, J. L. , S. E. Coen , and S. Bekker . 2021. “Anterior Cruciate Ligament Injury: Towards a Gendered Environmental Approach.” British Journal of Sports Medicine, September 55, no. 17: 984–990. 10.1136/bjsports-2020-103173. 33692033

[ejsc70102-bib-0029] Premiership Women’s Rugby . 2024. About PWR | Premiership Women’s Rugby. Accessed, May 19, 2025. https://www.thepwr.com/about‐pwr.

[ejsc70102-bib-0030] Sale, K. J. E. , T. R. Flood , S. M. Arent , et al. 2023. “Effect of Menstrual Cycle and Contraceptive Pill Phase on Aspects of Exercise Physiology and Athletic Performance in Female Athletes: Protocol for the Feminae International Multisite Innovative Project.” BMJ Open Sport & Exercise Medicine, November 9, no. 4: e001814. 10.1136/bmjsem-2023-001814. PMC10679978 38022756

[ejsc70102-bib-0031] Saynor, Z. L. , A. Hassan , and F. Wilson . 2024. “Women’s Rugby as a Catalyst for Advancing Female‐Specific Science and Safety in Sport.” European Journal of Sport Science 24, no. 12: 1683–1687. 10.1002/ejsc.12212. 39539197 PMC11621377

[ejsc70102-bib-0032] Starling, L. T. , N. Gabb , S. Williams , S. Kemp , and K. A. Stokes . 2023. “Longitudinal Study of Six Seasons of Match Injuries in Elite Female Rugby Union.” British Journal of Sports Medicine, February 57, no. 4: 212–217. 10.1136/bjsports-2022-105831. 36428090

[ejsc70102-bib-0033] Todd, I. 2025. “Dual Careers: The PWR Athletes Juggling Working Alongside Playing.” RugbyPass. Accessed, October 29, 2025. https://www.rugbypass.com/news/dual‐careers‐the‐pwr‐athletes‐working‐alongside‐playing/.

[ejsc70102-bib-0034] Truong, L. K. , S. Bekker , and J. L. Whittaker . 2021. “Removing the Training Wheels: Embracing the Social, Contextual and Psychological in Sports Medicine.” British Journal of Sports Medicine, May 55, no. 9: 466–467. 10.1136/bjsports-2020-102679. 33184111

[ejsc70102-bib-0035] Williams, S. 2024. “Women’s Professional Rugby Injury Surveillance Project Report 2023‐24.” Accessed July 11, 2025. https://rpubs.com/sw356/WRISP_2023‐24.

[ejsc70102-bib-0036] World Rugby . 2024. “‘Let This Upward Trajectory Continue’ – How the Women’s Game Soared in 2023.” Accessed May 19, 2025. https://www.world.rugby/news/898361?lang=en.

[ejsc70102-bib-0037] Zambaldi, M. , I. Beasley , and A. Rushton . 2017. “Return to Play Criteria After Hamstring Muscle Injury in Professional Football: A Delphi Consensus Study.” British Journal of Sports Medicine 51, no. 16: 1221–1226. 10.1136/bjsports-2016-097131. 28246078

