# Supplementary material for: Performance and Wellbeing Research Priorities in Premiership Women's Rugby: A Delphi Study Including Players and Staff
Source: Eur J Sport Sci. 2025 Dec 23;26(1):e70102. doi: 10.1002/ejsc.70102 (PMC12724576; doi:10.1002/ejsc.70102)
Supplement: Supplementary file 1 — Supporting Information S1 [file EJSC-26-e70102-s001.docx]

# SUPPLEMENTARY

## Supplementary material 1

### Staff Questions

Please provide your email address so that we can include you in future rounds.

What is your current job title?

What is your ethnicity? (drop-down box)

What is your age (in years)? (drop-down box)

What is your highest level of education achieved (e.g. GCSEs, A-levels, undergraduate degree, master's degree, PhD)?

How many seasons have you worked in **Premiership Women’s Rugby**?

How many seasons have you worked in the **Red Roses**?

How many seasons have you worked in **another sports league** at an **academy / pathway** level?

How many seasons have you worked in **international rugby union**at an **academy / pathway** level?

How many seasons have you worked in **another sports league** at **a senior** **first team**level?

How many seasons have you worked in **international rugby union** at **senior first** **team** level?

### Players Questions

Please provide your email address so that we can include you in future rounds.

What is your primary playing position? (drop-down box)

What is your ethnicity?  (drop-down box)

What is your age (in years)? (drop-down box)

What is your highest level of education achieved (e.g. GCSEs, A-levels, undergraduate degree, master's degree, PhD)?

How many seasons have you played in**Premiership Women’s Rugby**?

How many seasons have you played in **another senior league**?

How many times have you been selected for an **international rugby union**match-day squad **pathway** level?

How many times have you been selected for an **international rugby union** match-day squad at a **senior first team** level?

### Staff and Players Priority Generation

In the following section, you will be asked to provide up to 10 research priorities in the areas of wellbeing and performance. Please provide as much detail as possible for each suggested research priority.

**WELLBEING RESEARCH PRIORITIES** 
 
**Please list up to 10 specific player wellbeing research priorities that you feel are important for Premiership Women’s Rugby.** 
 
**Research definition:** *The process of studying something to discover new information or reach a new understanding.*   
 
**Wellbeing definition:** *A positive state experienced by individuals. Similar to health, it is a resource for daily life and is determined by social, economic and environmental conditions. It encompasses quality of life, as well as the ability of people to contribute to the world in accordance with a sense of meaning and purpose (Adapted from WHO, (2021)). Components of wellbeing include Emotional (positive / negative emotional states e.g. happiness, stress, anxiety), Mental (e.g. purpose, resilience, achievement), Social (e.g. relationships, social integration / acceptance) Physical (e.g. general physical health, injuries, financial and living circumstances) (Adapted from Giles, (2020)).* 
 
**For example:** an investigation into the stressors experienced by players in the Premiership Women’s Rugby.

**PERFORMANCE RESEARCH PRIORITIES** 
 
**Please list up to 10 specific player performance research priorities that you feel are important for Premiership Women’s Rugby.** 
 
**Research definition:** *The process of studying something to discover new information or reach a new understanding.*   
 
**Performance definition:** *Anything that contributes to how well a player or team can compete in match-play.* 
 
**For example:** an investigation into the transfer of tackle technique training to match scenarios.

## Supplementary table 1

**Supplementary table 1.** Higher-order research priorities that did not reach consensus, distribution of votes for priority across three categories, and the median priority (interquartile range, IQR).

| **Category** | **Higher-order research priority** | **Low priority (%)** | **Medium priority (%)** | **High priority (%)** | **Median priority (IQR)** |
| --- | --- | --- | --- | --- | --- |
| **Wellbeing theme** | | | | | |
| Medical | Understand the impact of female-specific factors (e.g., breast health, pregnancy, menstrual cycle phase or irregularity, hormonal contraceptive use) on wellbeing. | 2 | 30 | 67 | 4.0  (3.0 – 4.5) |
| Medical | Investigate the prevalence, severity and management of female health disorders (e.g., PCOS, endometriosis, urinary incontinence, menstrual irregularity). | 12 | 26 | 63 | 4.0  (3.0 – 4.0) |
| Fatigue and recovery | Investigate nutritional practices and the impact of nutritional interventions on player health and wellbeing. | 12 | 33 | 56 | 4.0  (3.0 – 4.0) |
| Psychology | Understand the impact of setbacks (e.g., injury, non-selection) on wellbeing, and any support mechanisms required. | 21 | 37 | 42 | 3.0  (3.0 – 4.0) |
| Medical | Investigate the prevalence, severity and management of general health disorders (e.g., REDs, IBS). | 21 | 40 | 40 | 3.0  (3.0 – 4.0) |
| Transitions | Understand the experiences (e.g., physical, social, financial, mental health) of retired players and the support mechanisms in place. | 19 | 42 | 40 | 3.0  (3.0 – 4.0) |
| Transitions | Understanding the effect of transitioning between environments (e.g., university to PWR, PWR to international, PWR to retirement). | 19 | 42 | 40 | 3.0  (3.0 – 4.0) |
| Psychology | *Understand the impact of performance profiling (e.g., aerobic capacity, body composition) on wellbeing. | 23 | 42 | 35 | 3.0  (3.0 – 4.0) |
| Psychology | *Investigate the impact of confidence or self-esteem on wellbeing. | 28 | 40 | 33 | 3.0  (2.0 – 4.0) |
| **Performance theme** | | | | | |
| External stressors | Investigate the impact of external stressors (e.g., financial status, team environment) on performance. | 12 | 26 | 63 | 4.0  (3.0 – 4.0) |
| Fatigue and recovery | Investigation of recovery practices (e.g., sleep, nutrition), and the impact of these on recovery and performance. | 9 | 28 | 63 | 4.0  (3.0 – 4.0) |
| Psychology | Investigate the factors that influence mental resilience. | 16 | 40 | 44 | 3.0  (3.0 – 4.0) |
| Match characteristics | Identify and compare PWR match and training characteristics. | 19 | 49 | 33 | 3.0  (3.0 – 4.0) |
| Transference | Investigate methods to maximise technical skill learning and subsequent transfer to match performance. | 26 | 42 | 33 | 3.0  (2.5 – 4.0) |
| Female-specific | Investigate the impact of kit specifically designed for females (e.g., boots, match jerseys) on performance. | 28 | 42 | 30 | 3.0  (2.0 – 4.0) |
| Player characteristics | *Investigate the prevalence of neurodivergence (e.g., autism, ADHD, dyslexia), the impact on performance, and any support mechanisms required. | 30 | 40 | 30 | 3.0  (2.0 – 4.0) |
| Transference | Investigate the transfer of technical training (e.g., collision skills, kicking skills) to match performance. | 23 | 49 | 28 | 3.0  (3.0 – 4.0) |
| Player characteristics | Describe the physical characteristics of players in the PWR, and explore which physical characteristics underpin successful technical performances. | 47 | 28 | 26 | 3.0  (2.0 – 3.5) |
| Transference | Investigate the transfer effect of physical and technical training interventions on performance. | 23 | 53 | 23 | 3.0  (3.0 – 3.0) |
| Match characteristics | Describe the technical characteristics of players in the PWR, and explore which technical characteristics underpin successful performances. | 30 | 49 | 21 | 3.0  (2.0 – 3.0) |
| Communication | Investigate effective methods of communication during match-play. | 37 | 44 | 19 | 3.0  (2.0 – 3.0) |
| **Injury theme** | | | | | |
| Injury risk | Investigate the association between training characteristics (e.g., contact training, volume of change of direction, overall training load) and injury. | 21 | 19 | 60 | 4.0  (3.0 – 4.0) |
| Injury risk | Investigate the association between nutritional status (e.g., micronutrient deficiency) and injury. | 7 | 40 | 53 | 4.0  (3.0 – 4.0) |
| Injury risk | *Investigate the association between sleep characteristics (e.g., duration, quality) and injury. | 16 | 33 | 51 | 4.0  (3.0 – 4.0) |
| Injury epidemiology | Investigate epidemiology of injury and concussion, and compare between player sub-groups (e.g., playing position, age, contract or employment status). | 12 | 40 | 49 | 3.0  (3.0 – 4.0) |
| Injury risk | Investigate the association between physical characteristics (e.g., lower limb strength, neck strength, body composition) and injury. | 7 | 51 | 42 | 3.0  (3.0 – 4.0) |
| Injury risk | Investigate the association between technical skills (e.g., tackle competence) and injury. | 14 | 44 | 42 | 3.0  (3.0 – 4.0) |
| *Denotes research priorities identified in Round 2. Priority rating: Low (1- very low, 2 – low), Medium (3 – medium), High (4 – high, 5 – very high). | | | | | |
